# Examining the Moderating Effects of Work Unit Size and Task Analyzability in the Relation Between Leader’s Communication Style and Leader-Member Exchange

**DOI:** 10.3389/fpsyg.2021.619060

**Published:** 2021-06-09

**Authors:** Ofelia Brown, Carmen Paz-Aparicio

**Affiliations:** ^1^Academic Department of Management, ESAN University, Lima, Peru; ^2^Institute of Entrepreneurship and Family Business, Universidad Carlos III de Madrid, Madrid, Spain

**Keywords:** leader-member exchange theory (LMX), leader’s communication style, work unit size, task analyzability, organizational communication, managerial communication

## Abstract

There is a consensus regarding the impact of the leader’s communication on the relationship with their followers and on the achievement of organizational outcomes. This study seeks to contribute to clarifying the impact that contextual factors have on the leader’s communication in order to know how leaders should adjust their communication style, depending on the job characteristics, to build high quality relationships with their followers. Therefore, the current research examines the moderating role of two context factors in the effectiveness of leaders’ communication in generating the leader-member relationship. Through a moderation analysis on a sample of 149 white-collar workers, this research study analyzes how *work unit size* and *task analyzability* interact regarding six dimensions of leader communication style in relation to LMX. Results suggest that the work unit size moderates the relationship between two dimensions of leader’s communication style (preciseness and verbal aggressiveness) and LMX. Specifically, the positive effect of preciseness on LMX smooths as the work unit size increases. The negative effect of verbal aggressiveness on LMX becomes more intense as work unit size increases. Furthermore, task analyzability moderates the positive relationship between emotionality and LMX for low levels of task analyzability. As a result, this study contributes by deepening on why leaders’ communicative behaviors can have favorable/unfavorable results in specific contexts and on how a leader can modulate his/her communication style according to the context, in order to improve the LMX. Implications are discussed.

## Introduction

Communication plays a crucial role in management (Christensen and Cornelissen, 2011; Taylor, 2011). There is a general consensus regarding the impact of a leader’s communication on the achievement of organizational outcomes (Robichaud et al., 2004; Johanson et al., 2014). Nevertheless, how leaders should adapt their communication style to different situations and work conditions is an issue that still needs to be studied in more detail. In recent decades, the need to incorporate contextual factors into the research of the relationship between variables has been recognized as an imperative to obtain more accurate results (Rousseau and Fried, 2001; Johns, 2006; Bamberger, 2008). This study seeks to contribute to clarifying the impact that contextual factors have on the leader’s communication in order to know how leaders should adjust their communication style, specifically depending on the characteristics of the work unit and the task, in order to build high-quality relationships with their followers.

According to the Leader-Member Exchange Theory (Dansereau et al., 1975; Graen and Schiemann, 1978; Liden and Graen, 1980), leadership is a relationship between a leader and a subordinate. The leadership relationship is created and maintained through day-to-day interactions in the executions of their roles (Fairhurst, 1993). From a communication perspective, the relationship is created through communicative interactions/exchanges to share the vision, establish goals, coordinate, inform, instruct, motivate, delegate, negotiate, gather opinions and suggestions, make participative decisions, provide feedback and coaching. Leader and followers use the available means depending on if the job is executed in physical or remote working, e.g., face-to-face, telephone conversations, text messaging, physical and virtual meetings, emails, chats, videoconferencing, social media, and other formal and informal channels in the organization.

In the same line, leaders create a different relationship with each subordinate. With some collaborators, it can become a high-quality relationship that is characterized by high levels of respect, trust, and mutual support, while with others, it is a medium or low-quality relationship (Graen and Uhl-Bien, 1995). To achieve the objective of deepening in the understanding of how communication contributes to the quality of the leadership relation, a multidimensional model of the leaders’ communication style (de Vries et al., 2009, 2010) has been used. A multidimensional model can allow for the identification of the communicative behaviors that should be adjusted depending on the context. According to de Vries and colleagues (de Vries et al., 2011; Bakker-Pieper and de Vries, 2013), the leaders’ communication style encompasses 24 facets in six dimensions (*expressiveness, preciseness, verbal aggressiveness, questioningness, emotionality and impression manipulativeness*), which are always somehow present and constitute the leader’s personal and unique communication style.

This study seeks to contribute to the understanding of the relationship between leader communication style and LMX incorporating two context variables. This study analyzes if work unit size (WUS) and task analyzability (TA) are contextual variables that the leader should consider to adapt his/her communication style to achieve high LMX relationships with their followers. Nowadays, it is common for frequent changes in the business world (mergers and acquisitions, restructuring, rightsizing). Workgroups, goals, and tasks are transformed, and leaders must adjust their leadership styles, which could, at the same time, require adjustments to their communication style. The *work unit size* can be a relevant contextual variable for communication studies as a leader’s resources, like time and support, could change and affect the quality and frequency of communication exchanges as the work unit grows or shrinks. In the same way, *task analyzability* (Wood et al., 1987; Campbell, 1988) has been included in the study. A higher or lower degree of task structure may mean, for the subordinate, different levels of difficulty and may affect the requirements of the leader’s communication. Unstructured tasks, due to their high ambiguity, may increase the need for greater information, support or feedback from the leader, whereas structured tasks, because they could be demotivating, may increase the need for contact that is more frequent, humor and emotion in the leader’s communication.

The paper is organized as follows: We first introduce the theoretical framework used to test our hypotheses and to explain our contribution. We then describe the data, the variables, and the method used. The following section describes the main results. Finally, we discuss the main findings and describe the study’s contributions and implications, its limitations, and topics for future research.

### The Role of Context

Few concepts are as complex and challenging to define as context. It is an amorphous concept in that it encompasses the relevant theory for the study of the phenomenon itself, everything that surrounds it, and the temporal conditions in which it occurs (Bamberger, 2008: 839). In other words, the context in which a leader exercises leadership includes both the opportunities and the restrictions (Johns, 2006), which must deal in the day-to-day. Contextual factors could explain why some communication behaviors of leaders are appropriate in certain circumstances and unfavorable in others. Conceptually, the environmental conditions act as forces that can affect the results, such as changing the causal direction between the variables, reversing the signs of the relationships, explaining curvilinear effects or precarious relationships, or even jeopardizing the validity of the results (Johns, 2006).

In the study of a phenomenon, different levels of context are distinguished. Johns (2006) proposes two levels of analysis: a broad dimension (omnibus context) and a particular dimension of factors that shape behaviors or attitudes (discrete context). The *broad context* is concerned with the occupation of those who make up the team, the location, the time, and the rationalization; while the *discrete context* comprises the specific situational variables that directly influence behavior or moderate the relationship between variables related to the task (e.g., autonomy, structuring, variety, interdependence, complexity, accountability, resources), the social context (e.g., density, social structure, social influence) and physical aspects (e.g., infrastructure, temperature, lighting). Joshi and Roh (2009), in their meta-analysis on the role of contextual factors in the investigation of team diversity, present a broad relationship of contextual factors considered by researchers, including characteristics associated with the task, the leader, and the team.

In order to answer the question regarding which contextual factors are relevant for our study, it is necessary to consider their nesting (Griffin and Mathieu, 1997; Perlow et al., 2004; Taylor, 2011). The LMX happens inside a work unit, which develops a specific function or project and has certain characteristics. These make it appropriate to consider the level of the work unit as the one immediately above, using a multilevel approach to sensitize the conclusions regarding the relationship between communication and LMX.

Among the diversity of variables at the work unit level found in the literature (Joshi and Roh, 2009) that could influence the relationship between the leader’s communication style and the LMX, our interest is focused on the size of the work unit and the complexity of the task, both discrete context variables (Johns, 2006). On the one hand, the WUS of the group has been incorporated because: a) the quality and frequency of communication contacts can be affected as the work unit grows and b) the current business dynamics make frequent changes in organization structure due to restructuring processes, mergers and acquisitions, project implementation, expansions or payroll reductions, which leads to variations in the size of the work units. Leaders find themselves in the situation of adjusting in their leadership styles according to the group’s size, which may be reflected in their communication style. On the other hand, the structuring of the task has been incorporated into the study as a contingent factor because structured (non-challenging) or unstructured (challenging) tasks demand different levels of effort from the subordinate that may affect the communication requirements with their leader. The following sections will discuss how these two variables could influence the relationship between leader’s communication on the LMX.

### Communication Style and LMX

The basis in this study is the relationship between the leader’s communication style and the quality of the relationship between the leader and the subordinate. The Leader-Member Exchange Theory (LMX) (Graen and Uhl-Bien, 1995) explains leadership as a dyadic relationship. Leaders do not relate to all their subordinates in the same way but create relationships of varying levels of quality with each one. LMX is built through day-to-day exchanges in which communication is the mechanism of interrelation and when the LMX is high, the relationship exhibits respect, trust and mutual obligation (Graen and Uhl-Bien, 1995). To achieve that, a leader’s communication characteristics must contribute to being perceived as a competent communicator, capable of exercising interpersonal influence through interactions (Bambacas and Patrickson, 2008; Johanson et al., 2014). To be perceived as a competent communicator, a leader should expose communicative behaviors that have been extensively studied in the management literature.

This study proposes that the communication style of the leader influences the LMX. An epistemological issue may arise regarding whether the leader’s communication style determines the leader-follower relationship (LMX) or whether it is the LMX that determines communication. The leader’s communication is based on his/her lifelong background. From birth, the human being begins to develop his/her ability to interact with others through communication. Therefore, at the moment he/she becomes a leader brings lifelong communication habits and behaviors, which are used when he/she occupies a managerial position and ultimately influences his/her collaborators. We all have a communication style that distinguishes us from the others, and that has been created since childhood. Over time, this style accumulates the effect of variables such as age, life experience, personality, and character, among others (de Vries et al., 2011). This personal way of communicating explains the level of influence or persuasion, the quality of our human relationships, and, to a certain extent, whether or not we are perceived as a leader (Ruben and Gigliotti, 2016). The communication style involves the content of the messages and the way we deliver them. Subordinates and other organization members perceive us through our way of communicating, how we interact with them in different situations. Contrasting these perceptions with his/her mental model of leader (Lord et al., 2001) produces the acceptance or rejection of the leadership proposal.

This does not exclude that LMX may in turn influence the way the leader communicates, since the interactive nature of communication and its bidirectionality create the dynamics of mutual influence between both interlocutors (Lee and Kim, 2021). The leader can be influenced and react with a particular communication style, which in turn will influence the LMX. This dynamic can lead to a virtuous or vicious circle, which generates a strengthening or deterioration of the LMX. Being the leader in a superior hierarchical position than the subordinate, his/her power to influence is greater than that of subordinates to influence the leader. Nevertheless, although we consider this influence to be smaller, it does not mean that it does not exist.

The literature recognizes that there are different patterns in the leader’s communication according to the quality of the LMX (Omilion-Hodges and Baker, 2017). In situations in which the leader and subordinate achieve a high-quality LMX, frequent and timely exchanges of information, support and trust can be observed (Campbell et al., 2003). The leader provides supportive communication that, in turn, favorably impacts the subordinate’s dedication to working and facilitates the relationships among coworkers (Michael et al., 2005). Similarly, Mueller and Lee (2002) demonstrate that in high-quality LMX relationships, the leader’s communication is characterized by openness, trust, empathy and attention to the employees, who receive enough valuable information to carry out their work. In contrast, in cases of low-quality LMX, communication patterns are characterized by antagonism and adversity (Fairhurst and Chandler, 1989).

The communication characteristics that influence interpersonal relationships, in general, are well-researched (McCroskey et al., 2001). The communication characteristics that have been studied are, for example, assertiveness (Deluga and Perry, 1991), Machiavellianism (Teven et al., 2006), defensive tendencies (Baker, 1980; Becker et al., 2005), communication apprehension (Teven et al., 2006; Madlock et al., 2007) and verbal aggressiveness (Infante et al., 1992; Martin and Anderson, 1998).

There is a gap in the literature about how leaders should modulate their communication in different contexts (Omilion-Hodges and Baker, 2017; Jian and Dalisay, 2018). In order to contribute, this study uses an integrated model to measure the leaders’ communication style (de Vries et al., 2009, 2010; Bakker-Pieper and de Vries, 2013) that identify 24 facets organized in six dimensions. From a contingency approach, we propose that the leader may and should modulate the communication style for achieving better results in his/her leadership. Workers perceive their leader through his/her behaviors and the integrated model addresses the issue using a multidimensional perspective based on observable communicative behaviors.

A “leader communication style” is defined as “a distinctive set of interpersonal communicative behaviors geared toward the optimization of hierarchical relationships in order to reach certain group or individual goals” (de Vries et al., 2010, p. 368). De Vries and colleagues report the multidimensionality of the construct, in which they identify 24 facets, organized into the following six dimensions: *expressiveness, preciseness, verbal aggressiveness, questioningness, emotionality, and impression manipulativeness.* These are traits (not types) of the leader’s communication style and are always present in the way a person communicates. Their particular combination constitutes our typical and personal way of communicating.

#### Expressiveness

(Facets: talkativeness, conversational dominance, humor and informality). This dimension includes the leader’s predisposition to talk, in a frequent and eloquent way. For example, this trait is perceived when the leader acts in a casual and informal way, without creating unnecessary barriers, showing an open, non-conflictive attitude, good humor, and with a suitable level of conversational adroitness toward all kinds of interlocutors. Moreover, it is perceived in the leader’s predisposition to express his/her ideas and lead the discussion, determining the topics to be discussed.

#### Preciseness

(Facets: structuredness, thoughtfulness, substantiveness, and conciseness). The leader shows accuracy in the communication of thoughts, through a logical and well-organized sequence of the different parts of the messages. The leader structures the message in a concise and pertinent manner, and without dwelling on matters that are irrelevant to the purpose. The leader thinks carefully before saying something, choosing words with care and weighing the answers before expressing them. The messages are concise and involve important topics, avoiding trivial ones.

#### Verbal Aggressiveness

(Facets: anger, authoritarianism, derogatoriness, and non-supportiveness). This trait includes the open expression of displeasure or anger about issues or people. The leader’s communication style manifests a low level of respect for others’ opinions. Discourages dialog, humiliates, hurts feelings and makes others look like fools. The subordinates feel that the leader neither gives attention to them nor understands their problems or needs and that he/she offers little support and treats people in a distant and cool way.

#### Questioningness

(Facets: unconventionality, philosophicalness, inquisitiveness, and argumentativeness). This trait is shown when the leader stimulates discussions about the future, engages in philosophical conversations and solicits different points of view. Usually the leader uses questions to stimulate others to delve into a topic, seeking to challenge the team intellectually. The leader likes to promote healthy debate and exchange of opinions, through the open discussion of new ideas, including wild or bizarre ones.

#### Emotionality

(Facets: sentimentality, worrisomeness, tension, and defensiveness). The leader manifests high levels of sentiment, including emotions and moods, when communicating during conversations. The leader tends to show concern, anxiety, and stress about daily routine issues. As a mechanism for protecting against dissenting opinions or criticisms, the leader copes poorly with critical remarks.

#### Impression Manipulativeness

(Facets: ingratiation, charm, inscrutableness, and concealingness). This trait refers to communicative behaviors related to the leader’s concern of controlling or manipulating others’ opinions. The leader expresses opinions different from what he/she really thinks, hiding the true way of thinking or information in order to appear better and gain acceptance from third parties, including boasting about ideas or achievements. He/she can show gentle, kind and courteous behavior, even with people or situations that he/she dislikes, in a polite and politically correct way.

The six dimensions are part of the personal communication style and we expect they correlate with LMX. According to the literature, expressiveness, preciseness, questioningness and emotionality benefit the leader-member bond (Campbell et al., 2003; Michael et al., 2005; Johanson et al., 2014; Lloyd et al., 2017; Jian and Dalisay, 2018), while verbal aggressiveness (Fairhurst and Chandler, 1989; Bakker-Pieper and de Vries, 2013) and impression manipulativeness affect it negatively (Teven et al., 2006).

### Work Unit Size (WUS)

The size of a work unit refers to the number of positions formally grouped within a single unit reporting to the same superior. It is a structural variable that is taken into consideration in decisions regarding work unit configurations (Mintzberg, 1980) because it moderates their effectiveness (Campion et al., 1996). The WUS should be adjusted based on the characteristics of the tasks. A team that is too large could be difficult to manage and could cause its members to lose interest due to lack of individual participation, while the opposite —teams with too few members— could experience too much workload, and the work unit could lack the resources necessary to complete the tasks and achieve its goals (Dyer et al., 2013). From another perspective, when the activities carried out in the work unit are standardized and normalized, the units may have a greater number of job positions because processes and results are well defined and require less direct supervision. However, when activities require coordination among members and constant adjustments, the units tend to be small because more communication is required and this may only occur if the work unit is small (Mintzberg, 1980).

The results reported in the literature about the impact of WUS on LMX are contradictory (Schyns et al., 2010). Green et al. (1996) studied the relationship between demographic and organizational variables on LMX (one of them is the size of the work unit). They concluded that the WUS is negatively related to the LMX quality, confirming similar results found in a previous study carried out by the same authors in 1983, in the branch offices of a bank. However, Cogliser and Schriesheim (2000) came to different conclusions; they did not find support for their hypothesis that WUS has a significant relationship with LMX. The relationship they found was negative but not significant. Our study seeks to contribute to this vein to determine if WUS influences the creation and maintenance of high-quality relationships with subordinates through communication.

According to Keyton and Beck (2008), when communication is not adapted to WUS, problems may arise, as the time a leader has to interact with each team member is reduced. Because the leader’s time is a finite variable, the greater the WUS, the less time the leader will have to interact with each one. It is expected that the fewer resources a leader dedicate to each member; this will affect the communication characteristics, such as the duration, content, channel employed and communication climate. This effect on communication will affect the LMX quality. We thus propose the following hypothesis:

***Hypothesis 1 (H1):*** *WUS moderates the relationship between the leader’s communication style and LMX.*

Variations in the size of the work unit may mean that leaders must adapt their communication style to a given situation, redistributing their resources of time and attention to satisfy the workers’ needs and to not affect the work unit’s performance (Keyton and Beck, 2008). Expressiveness is the dimension that measures the talkativeness, conversational dominance, informality, and humor of the leader. This trait is perceived when the leader communicates in an open, casual, informal, and frequent way without creating unnecessary barriers. His/her expressiveness is positively related to LMX (de Vries et al., 2010; Brown et al., 2019). Considering that leaders have finite resources of time and availability to distribute their attention to all their workers, a larger WUS can reduce the opportunity and quality of contact with all the group members. Workers may perceive the leader’s communication expressiveness as insufficient to construct a high-quality LMX relationship and/or obtain complete information regarding the work unit’s objectives and goals. We propose the following hypothesis:

***Hypothesis 1a (H1a):*** *WUS moderates the positive relationship between expressiveness and LMX in such a way that the relationship is weakened when WUS is higher.*

The preciseness in the leader’s communication style is related to the accuracy in the delivery of thoughts, through a logical and well-organized message. The message is presented in a concise, direct and relevant structure, without irrelevant content. Preciseness is positively related to LMX (de Vries et al., 2010; Brown et al., 2019) since it favors the understanding of the task, the objectives, the expectations and the vision of the boss. In small groups, the leader’s message is received directly by the workers who have an even greater chance of obtaining immediate feedback from the leader himself. In groups with a greater number of collaborators, it is not possible for the leader to communicate one by one, so it is possible that the message has to be retransmitted by a third person or that the message is received through deferred channels. In addition, it may happen that the worker is not able to get direct feedback from the boss if he has not understood something. The positive impact of precision on the LMX in the latter case may be affected, reducing the intensity of the relationship as the group grows. We propose the following hypothesis:

***Hypothesis 1b (H1b):*** *WUS moderates the positive relationship between preciseness and LMX in such a way that the relationship is weakened when WUS is higher.*

The verbal aggressiveness dimension is recognized by the literature as a destructive feature of communication that dramatically affects interpersonal relationships due to its potential to damage the receiver’s self-concept and psychological wellbeing (Infante et al., 1992). When the size of the unit increases, leaders are faced with a greater work demand, which makes their leadership style more impersonal, autocratic and strict and reduces their opportunities for interacting with their subordinates (Hemphil, 1950). Schyns et al. (2012) found that in large groups, to build high-quality LMX relationships, leaders needed to show high levels of kindness and politeness, characteristics that are the opposite of verbal aggressiveness. Therefore, the following hypothesis is proposed:

***Hypothesis 1c (H1c):*** *WUS moderates the negative relationship between verbal aggressiveness and LMX in such a way that the relationship is strengthen when WUS is higher.*

The questioningness dimension is displayed in inquisitive characteristics, which stimulate the discussion of issues, proposing the exchange of opinions and witty, unconventional and curious expressions. Large WUS reduces the possibilities of close interactions of all the members with the leader (Schriesheim et al., 2000; Hare and Hare, 2003). Additionally, workers tend to increase the expression of their opinions when the unit is smaller rather than larger (LePine and Van Dyne, 1998). At the same time, even if a leader intends to foster everyone’s participation, the time availability could reduce the frequency and possibility of involvement of all members. Therefore, we propose that:

***Hypothesis 1d (H1d):*** *WUS moderates the positive relationship between questioningness and LMX in such a way that the relationship is weakened when WUS is higher.*

Emotionality, in leaders’ communication, is measured through the exteriorization of behaviors related to worry, anxiety and stress. It is expected that these behaviors can interact with WUS so that if a group is small, closeness with the leader can help the group members to satisfactorily understand and accept the leader’s emotionality. In contrast, in large groups, there is a greater physical and psychological distance between the subordinates and leader (Schyns et al., 2010), and it is possible for group members not to have access to all the information about the work unit that allows them to interpret the leader’s emotionality, thus leading to their rejection. Therefore, the following hypothesis is proposed:

***Hypothesis 1e (H1e):*** *WUS moderates the positive relationship between emotionality and LMX in such a way that the relationship is weakened when WUS is higher.*

The impression manipulativeness dimension, also known as Machiavellianism or relational manipulation, comprises behaviors in which the leaders’ messages are neither open nor transparent and instead, hide their true thoughts or intentions in order to achieve acceptance or to ingratiate themselves with their interlocutors. These behaviors affect the LMX negatively (Bakker-Pieper and de Vries, 2013) because they generate distrust in the leader and erode the perception of ethics and integrity that are required from a leader. In small groups, in which the frequency of contact is higher and there is a smaller psychological distance, it can be expected that greater intensity of this dimension does not cause, *per se*, the negative impact on LMX. On the other hand, in large groups, due to a greater psychological distance and a smaller possibility for the leader and subordinate to know each other, a higher level of manipulation would affect the LMX more intensely. Therefore, the following hypothesis is proposed:

***Hypothesis 1f (H1f):*** *WUS moderates the negative relationship between impression manipulativeness and LMX in such a way that the relationship is strengthen when WUS is higher.*

### Task Analyzability (TA)

The characteristics of the task may affect the work team’s performance and deserve the ample attention that the literature has paid them (Loher et al., 1985; Fried and Ferris, 1987). Task analyzability has been studied for its ability to explain phenomena at both the individual level and the group level (Wood et al., 1987; Campbell, 1988).

Separating the task from its doer, Wood (1986) identified the following three components of tasks: the resulting product, the required information, and the acts necessary for carrying it out. Moreover, he derived the following three dimensions, to measure its complexity: First, component complexity (as a function of the number of actions necessary to carry out the task); Second, coordination complexity (making reference to the relationship between information-acts-product to execute it, which involves times, frequencies, intensity, and location); and third, dynamic complexity (relating to the change through time which the actions and information suffer, which obligates the doer to adapt). Based on this, task complexity can be determined from an objective perspective, independent of who performs it, and it is directly related to its attributes, i.e., the accessibility of information, diversity of information and rate of change (Campbell, 1988). From this point, tasks may be classified based on the following four characteristics: first, the presence of multiple paths to obtain the product or result; second, the presence of multiple desired results; third, the presence of interdependencies in conflict between the paths and various desired results (the achievement of one result enters into conflict with the achievement of another desired result); and fourth, the presence of uncertainty (limited information) or possible connections between multiple paths and results (Campbell, 1988).

Therefore, measuring the complexity of a task is not easy, given that although attributes of the task itself can determine its complexity objectively, an observer, who will contribute his point of view based on his own perception, experience, and knowledge, should evaluate and grade it. Searching for objectivity in the measurement of complexity, Withey et al. (1983) developed an instrument to measure complexity using two dimensions. The first refers to the number of exceptions that a worker must make while carrying out the task, which is equivalent to the task’s *variety*, which is expressed in the frequency of unexpected or different events that occur in the conversion process. When the number of exceptions in high, the worker cannot predict possible problems, and the tasks become unique. When few exceptions occur, the task is repetitive. The second characteristic is the level of *structure* (*analyzability*) of the task. When a work process is structured, it can be understood as a sequence of previously identified steps known to the worker, which can be followed as a computational process. In contrast, when a task is not very structured (low analyzability), the sequence cannot be established objectively, which means that the worker must spend time thinking about how to carry out the task or solve the problem, as there are many paths for achieving it and many potential results (Withey et al., 1983). Highly varied tasks that are not very structured are the most challenging for workers.

The intensity of the challenge of a task is a function of the variations in the perception of the subjects who must carry it out, and this perception is reflected in the biases an individual has, based on his or her experience, familiarity with the task, frames of reference and attitudes (O’Reilly et al., 1980). This study has not included the *variety* dimension because we consider that analyzability includes variety in some way. When variety is low, the worker performs the same tasks repeatedly since there are no new tasks. By carrying out the same tasks, the worker knows the steps to carry them out, which is equivalent to the structuring being high and is able to perform them in a computational process. From the opposite perspective, a wide variety of tasks may be equivalent to the worker having to face different and probably unfamiliar tasks, which requires a lot of thinking about how to do the tasks and effort to find ways to solve them. What is considered relevant is the level of analyzability because this will determine the level of challenge a worker faces when dedicating effort to finding different ways to resolve the task, as less structured tasks can have multiple ways to carry them out and multiple possible results (Withey et al., 1983).

This study aims to answer whether TA moderates the relationship between the leader’s communication style and the LMX. A fundamental pillar of LMX theory is the emphasis on the relational process of leadership. In the execution of the tasks, the leader and the worker interact, and in this interchange, LMX is born and develops (Ferris et al., 2009). Leaders promote performance through goal-setting processes, which require reciprocal interactions between leaders and followers (Locke and Latham, 2002). The leader provides information on the task to be carried out, clarifies the expected results, provides the resources, support, and feedback. The worker must perform the task using his/her knowledge, experience, skills, motivation, and efforts to achieve the objectives. The LMX theory recognizes that it is through different types of exchanges that leaders differentiate the way they interact with their subordinates (Dansereau et al., 1975).

The literature recognizes that leaders adapt their leadership styles to the characteristics of the task and those of their subordinates (Dunegan et al., 2002; Dóci and Hofmans, 2015). Leaders can be assumed to have the knowledge, experience, and motivation to guide their subordinates successfully. However, Dóci and Hofmans (2015) propose that leaders activate different cognitive mechanisms depending on situational circumstances. Based on the literature, we propose that the task can be a variable that activates these different leadership mechanisms since the studies carried out on its role as an antecedent, consequence, and moderator have provided evidence of its impact on the worker and leadership.

The task is one dimension of core job design that impacts the psychological state of individuals by increasing the experienced meaningfulness of the work and explaining motivation, performance, and satisfaction (Hackman and Oldham, 1976). Martin et al. (2016) report a meta-analysis that examines the relationship between LMX quality and a multidimensional model of work performance, in which task dimension as a dependent variable is positively related to LMX. Additionally, they report that trust, motivation, empowerment, and job satisfaction mediate the relationship between LMX and task.

Dunegan et al. (1992) studied the moderating role of task complexity in the relationship between LMX and performance. They report that when the task challenge is either very high or very low, the relationship between LMX and performance is higher. However, when the task challenge is moderate, the relationship between LMX and performance is not significant. They supplemented their studies on the moderating role of task characteristics in a later study (Dunegan et al., 2002). They found evidence of the moderating role of three characteristics of the task: role conflict (inconsistent or contradictory assignments or obligations), role ambiguity (uncertainty about job duties and responsibilities), and intrinsic task satisfaction (person’s sense of connection and compatibility with a task) on the relationship between LMX and performance.

Considering the association between leadership and the task, the leader might need to adapt his/her communication style according to the characteristics of the task to achieve the expected results and the adequate performance of the worker. There is a gap in the literature regarding the moderating role of task complexity in the relationship between the leader’s communication and the LMX.

When workers are required to perform highly uncertain or challenging (less structured) tasks, they expect the leader to clarify and communicate the necessary information adequately; define the goals, products, and expected results; and provide support, which will contribute to success. On the contrary, if subordinates perceive that a task is not very demanding or feel that their skills and knowledge are sufficient to carry out the task, the leader’s clarifications could be considered unnecessary, controlling, demotivating and unsatisfactory (House, 1996). The characteristics of the task and the worker’s level of professionalism/mastery could make unnecessary the leader’s intervention (Kerr and Jermier, 1978; Howell and Dorfman, 1981) and affect communication requirements.

Michael et al. (2005) report that supportive supervisor communication influences LMX, affecting contextual and task performance. Their results suggest that when supervisors show their employees consideration, respect, and support through their communication exchanges, higher LMX are likely. Furthermore, the quality of the relationships that subordinates have with their supervisors influences their job dedication and interpersonal facilitation behaviors. Supportive supervisor communication creates an overall supportive environment and relationship quality that translates into higher employee contextual and task performance (Michael et al., 2005).

A part of the workers’ perception regarding task significance may be explained by the leaders’ influence through their messages. The perception of leaders can influence the perception of workers regarding the characteristics of the task (Griffin, 1981). Leaders can influence how workers perceive and interpret their work context, how the workers assess task significance and how involved they become in their tasks through their actions and verbal and non-verbal messages (Shamir et al., 1993). They personify their mission and vision, just as they frame through their messages the ideological content, values and intellectual reasoning, demonstrating the mental frame that inspires and guides them. Based on the literature, it can be expected that the leaders’ communication behavior influences the workers’ perceptions and values when facing structured or less structured tasks, which leads to the following hypothesis:

***Hypothesis 2 (H2):*** *TA moderates the relationship between the leader’s communication style and LMX.*

This study seeks to go beyond the analysis of the moderating effect of TA on the communication style-LMX relationship, by determining which dimensions of the leaders’ communication style are, that moderate the mentioned relationship. More leader intervention is necessary when workers face less structured tasks than when they face structured tasks (House, 1996). Leaders can help subordinates increase their self-esteem and self-worth through the communication of trust and of high-performance expectations (Shamir et al., 1993), and therefore, it is proposed that leader expressiveness can contribute to helping and clarifying for the unstructured task and improve the LMX quality.

***Hypothesis 2a (H2a):*** *TA moderates the positive relationship between expressiveness and LMX in such a way that the relationship is strengthen when the task is less structured.*

Preciseness in the leader’s communication style is exteriorized in the ability to structure messages concisely, clearly and professionally. No literature has been found that has studied the relationship between the preciseness of the leaders’ communication and TA. Subordinates simply require the leader to clarify the mission and vision, along with instructions for their execution (House, 1996), and therefore, it can be estimated that the ability of leaders to articulate their messages with preciseness improves the LMX quality more when tasks are not structured, as preciseness reduces ambiguity.

***Hypothesis 2b (H2b):*** *TA moderates the positive relationship between preciseness and LMX in such a way that the relationship is strengthen when the task is less structured.*

The verbally aggressive behavior of leaders toward their subordinates has been studied by Infante and Gorden (1985), who indicate that when less verbal aggressiveness is perceived in a leader, subordinates will be more open and prone to express their ideas and debate about what should be done and how it should be done. Given that less structured tasks offer a greater challenge, that situation could create a climate of occupational stress (Karasek, 1979), which sets off verbally aggressive behavior in leaders. According to Dóci and Hofmans (2015), leaders’ behaviors tend toward authoritarianism and antagonism when they face tasks that they perceive as difficult to achieve, because of a loss of psychological resources. It can be expected that, when facing structured tasks, the relationship between verbal aggressiveness and LMX continues to be negative, but the intensity of the relationship will increase when the worker faces less-structured tasks. For tasks that are more challenging for the worker, subordinates can perceive leader aggressiveness as a lack of understanding and a lack of trust in their abilities, and this can contribute to higher occupational stress. It can also affect the workers’ psychological wellbeing and, consequently, relates more negatively to LMX. Therefore, the following hypothesis is proposed:

***Hypothesis 2c (H2c):*** *TA moderates the negative relationship between verbal aggressiveness and LMX in such a way that the relationship is strengthened when the task is less structured.*

Questioningness manifests itself in communication behavior in which leaders show their keen, inquisitive and curious thoughts by looking for unconventional solutions, stimulating open discussion and promoting participation. These behaviors improve the LMX quality (Bakker-Pieper and de Vries, 2013) and are associated with the intellectual stimulation dimension of transformational leadership (Bass et al., 2003). When workers must carry out structured tasks, following a pre-established and well-known sequence, a greater or lesser degree of questioningness in the leader communication style would not influence the LMX. However, for unstructured tasks, subordinates could require a leader to interact with them to stimulate the search for alternatives and promote dialog to find optimal solutions. As transformational leadership theory states, leaders who, through their messages, exteriorize a participative search for methods and results that go beyond conventional methods and results are perceived favorably, which in turn favors the LMX. Thus, we propose the following hypothesis:

***Hypothesis 2d (H2d):*** *TA moderates the positive relationship between questioningness and LMX in such a way that the relationship is strengthened when the task is less structured.*

Emotionality in the leader communication style is associated with behaviors that exteriorize the leader’s feelings, emotions, moods, worry, tension, and anxiety when facing an occupational challenge. Through verbal and non-verbal messages, leaders create ideological frames and shape the workers’ assessment of task significance (Shamir et al., 1993). From there, workers can perceive the leaders’ assessment of task relevance through those leaders’ exteriorization of emotionality. When the task is unstructured, higher levels of emotionality may favor the LMX, because they could be interpreted as the task value and significance the leader assigns to the task. Therefore, the following hypothesis is proposed:

***Hypothesis 2e (H2e):*** *TA moderates the positive relationship between emotionality and LMX in such a way that the relationship is strengthened when the task is less structured.*

As previously indicated, impression manipulativeness, which is associated with Machiavellianism, comprises communication behaviors that mask the true thoughts or intentions of the leader, who uses it to gain acceptance or ingratiation with others. These behaviors are negatively related to LMX (Bakker-Pieper and de Vries, 2013) because they generate distrust and take away from the perception of ethics and integrity, which are basic requisites of leadership (Ciulla, 2005). When a task is less structured and challenges workers, their resources of attention, time and interest are concentrated on the task, and so a higher or lower level of this trait would not affect the LMX. However, the negative effect will be stronger when the worker carries out highly structured tasks. When facing unchallenging tasks, the subordinates have more time and attention resources to dedicate to other issues. If they perceive leaders as acting with the intention to manipulate, they will feel greater rejection and distrust for leadership, causing the LMX to deteriorate. This leads to the following hypothesis:

***Hypothesis 2f (H2f).*** *TA moderates the negative relationship between impression manipulativeness and LMX in such a way that the relationship is strengthened when the task is more structured.*

**FIGURE 1:**
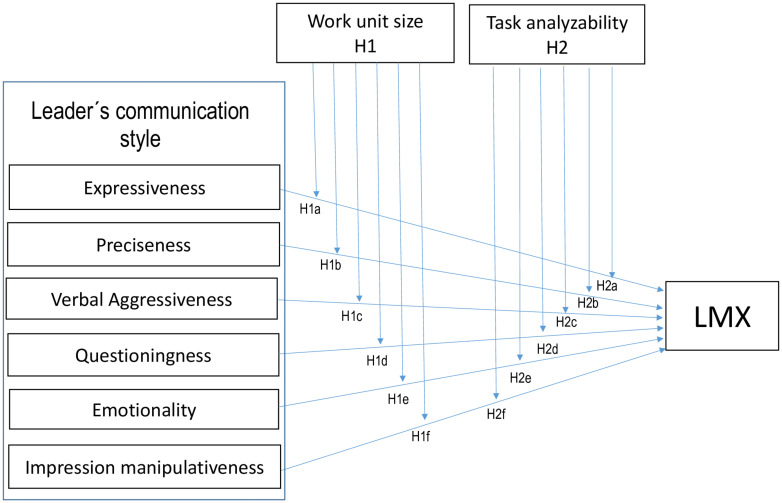
The research model.

Figure 1 shows the suggested research model, which represents the moderating effect of WUS and TA on the relationship between the leader’s communication style (expressiveness, preciseness, verbal aggressiveness, questioningness, emotionality, and impression manipulativeness) and LMX.

## Materials and Methods

### Database

The database was built through the participation of 149 working professionals contacted as students in 18 classrooms in postgraduate programs at the ESAN School of Business in Lima, Peru. The survey was distributed in paper and took approximately 30 min to complete. Originally, 279 subjects responded to the survey about their perception of their leader’s communication style and the characteristics of the group and the task they perform. Later on, there was a filtering process in which inconsistent or incomplete surveys were discarded and subjects were selected considering time under the command of the same leader.

The final sample was constructed with subjects who have completed 12 months under the command of the same leader to ensure that the leader-follower relationship has been able to go through the stages of its evolution until it reaches maturity and stability. According to Graen and Uhl-Bien (1995), the LMX goes through evolutionary stages, during which the communicative exchanges could vary.

At the beginning of the relationship, they both act as “strangers”. The relationship develops in the formal framework of hierarchical dependency, with a transactional nature (I give something-you give something) and contractually. Then, either party makes an “offer” to develop a better working relationship aimed at the subordinate’s career development, which once accepted encourages the duo to move to the second phase: “known,” with more frequent exchanges, begin to share more information and resources, both personally and in employment matters. The next phase, of “mature association”, is recognized because the exchanges are highly developed, reciprocal, unpaid, on more extended periods, individuals count on each other in relationships of loyalty and mutual support. They are behavioral and emotional exchanges: mutual respect, trust, and an implicit obligation to grow as in a process. Not all leader-subordinate dyads are formed at the same rate of advancement, and some even remain in the “unknown” phase, do not evolve, and are defined as low-quality LMX relationships. To ensure that the dyads have had the opportunity to go through the different evolutionary stages of the LMX, this study’s sample was made up of subjects who have worked 12 months under the same leader’s command.

Another relevant characteristic of the sample is that this is a workgroup with a high level of education, as 81% report having completed a university degree: 26% of the respondents have obtained a master or doctoral degree and 55% have completed undergraduate studies. 18% indicate technical or high school studies. About the job, 48% of the respondents are assistants or analysts and 52% are managers; the respondents have an average of 12 years of work experience. They work for private companies (76%), governmental entities (16%), socially owned enterprises (0.07%) and mixed private-public organizations (8%). Regarding the sociodemographic characteristics of the respondents, 69% are male, between 21 and 69 years of age, with an average age of 36 years. Finally, 97% are Peruvian citizens.

### Instrument

The instrument was built of items to measure the variables to be studied and was translated into Spanish, submitted to a test-retest process and validated by a panel of three professional translators.

#### Leader Communication Style

The *Communication Styles Inventory* (de Vries et al., 2011) which was adapted for the subordinates to evaluate the communication style of their direct leaders, was applied. It is composed of 96 items, organized in 16 items for each of the six dimensions. Examples of the items are: “My leader always have a lot to say” (expressiveness); “He always express a clear chain of thoughts when argue a point” (preciseness); “When he feels others should do something for him, he asks for it in a demanding tone of voice” (verbal aggressiveness); “In discussions, he often put forward unusual points of view” (questioningness); “When he is worried about something, he finds it hard to talk about anything else” (emotionality); “In discussions, he/she sometimes express an opinion he/she do not support in order to make a good impression” (impression manipulativeness). The items were answered on a Likert type scale of five categories, in which 1 was equivalent to “totally disagree” and 5 was equivalent to “totally agree.” The reliability of the instrument was measured by Cronbach’s alpha (Table 2).

#### Leader-Member Exchange

Graen and Uhl-Bien’s (1995) instrument of seven items were used. One example of the items: “My leader understands my job problems and needs”. The items were answered on a Likert type scale of five categories, in which 1 was equivalent to “totally disagree” and 5 was equivalent to “totally agree.” The scale shows a high level of reliability (α = 0.89).

#### Work Unit Size

An open question was included as follows: “Approximate number of people who report to the same leader as you do.”

#### Task Analyzability

It was measured using the four indicators of the instrument proposed by Dunegan et al. (1992), based on the work of Withey et al. (1983): “There is a clearly known way to do the major types of work I normally encounter.” “There is a clearly defined body of knowledge of subject matter that can guide me in doing my work.” “There is an understandable sequence of steps that can be followed in doing my work.” “I can actually relay on established procedures and practices to do my work”. The items were answered on a Likert type scale of five categories, in which 1 was equivalent to “totally disagree” and 5 was equivalent to “totally agree”. The level of reliability of the scale is α = 0.86.

#### Control Variables

The instrument included questions for the control variables of age and gender of the subordinate and of the leader.

In order to check the construct validity of the major variables, confirmatory factor analysis was carried out (Table 1). All measurements show a good model fit regarding CFI and TLI. According to Hu and Bentler (1999), values over.95 show a good model fit. In the case of LMX and questioningness, SRMR and RMSEA show slightly higher values than the acceptable threshold (0.05). Verbal aggressiveness and impression manipulativeness show slightly higher only in SRMR than the acceptable values (0.05). In spite of these, all items have factor loadings higher than.40 and CFI and TLI are adequate, therefore it is possible to conclude that communication styles, LMX and TA have a good model fit.

**TABLE 1:** Fit Indices of Leader-Member Exchange, Task Analyzability, and Leader’s Communication Style Dimensions.

Fit measures	LMX	Task analyzability	Expressiveness	Preciseness	Verbal aggressiveness	Questioningness	Emotionality	Impression manipulativeness
χ2	28.30**	2.88	35.90*	97.40	154.00***	37.60*	46.00	48.60*
df	14.00	2.00	23.00	104.00	90.00	24.00	54.00	32.00
CFI	0.99	1.00	0.98	1.00	0.98	0.98	1.00	0.98
TLI	0.98	0.99	0.96	1.00	0.98	0.97	1.01	0.98
RMSEA	0.06	0.04	0.04	0.00	0.05	0.05	0.00	0.04
SRMR	0.07	0.03	0.05	0.05	0.07	0.06	0.05	0.06

*p < 0.05; **p < 0.01; ***p < 0.001.

## Results

The data were processed and filtered to ensure information quality. Normal averages and standard deviations were observed. Statistical processing for the validation of the hypotheses was carried out using multiple hierarchical regressions with RStudio, Version 1.1.463. Regarding the risk of common method bias (Podsakoff et al., 2003), Harman’s single factor test was applied. It was found that forcing a single factor, it explains 21.88% of the variance. Additionally, 31 factors explain more than 77% of the variance. Because one single factor does not explain the majority of the variance, it is believed that the possibility of uniform method bias is not a limitation of this study. Table 2 presents the averages, standard deviations, and correlations between the variables of the study and the reliability indicators (Cronbach’s alpha) are included in the upper diagonal.

**TABLE 2:** Descriptive Statistics, Correlations, and Reliability.

		*Mean*	*s.d.*	*1*	*2*	*3*	*4*	*5*	*6*	*7*	*8*	*9*	*10*	*11*	*12*
1.	LMX	3,80	0,89	***(0.89)***											
2.	Work unit size	13,32	11,51	−0,08											
3.	Task Analyzability	3,83	0,88	0,33**	0,03	***(0.86)***									
4.	Expressiveness	3,11	0,61	0,08	−0,03	−0,13	***(0.72)***								
5.	Preciseness	3,62	0,66	0,65**	−0,07	0,28**	−0,03	***(0.88)***							
6.	Verbal Aggressiveness	2,60	0,79	−0,54**	0,04	−0,24**	0,32**	−0,66**	***(0.89)***						
7.	Questioningness	2,89	0,42	0,12**	0,13	−0,03	0,35**	−0,01	0,18**	***(0.77)***					
8.	Emotionality	2,64	0,76	−0,44**	0,07	−0,18*	0,26**	−0,64**	0,71**	0,25**	***(0.85)***				
9.	Impression Manipulativeness	2,59	0,77	−0,36**	0,00	−0,26**	0,38**	−0,46**	0,61**	0,36	0,62**	***(0.79)***			
10.	Age of Subordinate	35,91	8,66	0,08	0,09	0,08	−0,11	0,09	−0,12	−0,17	−0,03	−0,14			
11.	Gender of Subordinate			0,07	0,11	0,01	−0,04	0,08	−0,14	0,01	−0,15	−0,01	0,14		
12.	Age of Leader	46,50	9,46	0,18*	0,02	0,07	0,04	0,13	−0,08	−0,05	−0,13	−0,04	0,27**	0,00	
13.	Gender of Leader			0,15	0,24**	0,08	0,02	−0,02	0,00	0,08	−0,10	0,05	0,03	0,20*	0,18*

**. Correlation significant at the 0.01 level (bilateral). *. Correlation significant at the 0.5 level (bilateral). N = 149.

The LMX correlates with the moderator variable of *TA*, as do the following five leader’s communication style variables: *preciseness, verbal aggressiveness, questioningness, emotionality*, and *impression manipulativeness*. There is no sign of correlation with *expressiveness* and *WUS*. The *WUS* variable does not correlate with any variable in the study. The *TA* variable correlates with the following four dimensions of leader communication style: *preciseness, verbal aggressiveness, emotionality*, and *impression manipulativeness.*

Model 1 (Table 3) displays the results of the regression of the six dimensions with LMX without the moderator variables. This model is statistically significant, and the following four dimensions have significant betas: expressiveness, preciseness, verbal aggressiveness and questioningness. Impression manipulativeness and emotionality do not display a significant relationship. Overall, there is a positive relationship between LMX and expressiveness, LMX and preciseness and LMX and questioningness. Verbal aggressiveness has a negative relationship with the outcome. Both age and gender of leaders and of subordinates were used as control variables.

**TABLE 3:** The Moderating Effect of Contextual Variables on the Relationship between Leader’s Communication Style and LMX.

*Variables*	*Model 1*		*Model 2*	
	*B*	*e.t.*	*B*	*e.t.*
Age of Subordinate	0.00	0.01	0.00	0.01
Gender of Subordinate	−0.04	0.12	0.03	0.11
Age of Leader	0.01	0.01	0.00	0.01
Gender of Leader	0.31**	0.13	0.42**	0.13
Expressiveness	0.24**	0.10	0.22*	0.10
Preciseness	0.58***	0.12	0.46***	0.11
Verbal aggressiveness	−0.35*	0.11	−0.51***	0.10
Questioningness	0.30**	0.14	0.47**	0.14
Emotionality	0.10	0.11	0.07	0.12
Impression manipulativeness	−0.16	0.10	−0.13	0.10
Work unit size (WUS)				
WUS			−0.01*	0.01
WUS*Expressiveness			0.00	0.01
WUS*Preciseness			−0.03*	0.01
WUS*Verbal aggressiveness			−0.05***	0.01
WUS*Questioningness			0.03	0.02
WUS*Emotionality			0.02	0.01
WUS*Impression manipulativeness			−0.01	0.01
**Task Analyzability (TA)**				
TA			0.19**	0.07
TA*Expressiveness			0.15	0.01
TA*Preciseness			0.03	0.01
TA*Verbal aggressiveness			−0.15	0.01
TA*Questioningness			0.27	0.02
TA*Emotionality			0.29*	0.01
TA*Impression manipulativeness			−0.09	0.01
***R*^2^**	0.52		0.69	
**Adjusted *R*^2^**	0.49		0.62	
***F***	15.1***		10.30***	

*p < 0.05; **p < 0.01; ***p < 0.001.

To test Hypothesis 1, a multiple linear regression was carried out (Model 2). WUS shows a negative direct effect on LMX while controlling for communication style, TA, gender and age of leaders and subordinates. In addition, this variable shows a significant moderation effect in the relationship between preciseness, verbal aggressiveness, and LMX. Therefore, H1 is partially accepted as H1b and H1c are confirmed.

Results show that H2 is also partially accepted. TA shows a positive direct effect while controlling for communication style, WUS, gender, and age of leaders and subordinates. In this case, there is a significant moderation effect of TA in the relationship between emotionality and LMX, while controlling for the other communication styles, WUS, age and leader of leaders and subordinates. In that case, H2e is accepted.

**FIGURE 2:**
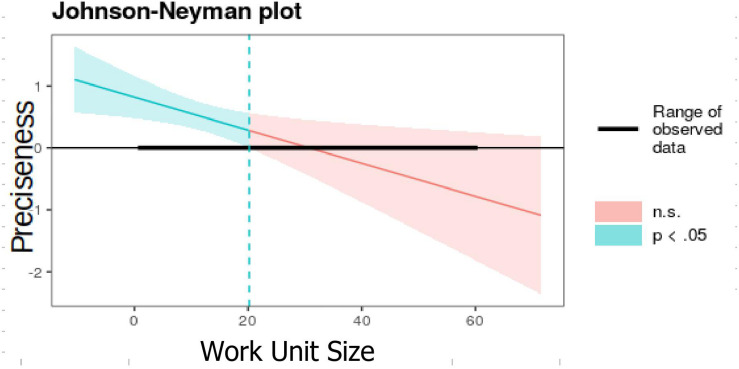
The moderating effect of WUS on the relationship between preciseness and LMX.

In order to further understand the moderating effect of WUS and TA in the relationship between communication styles and quality of the LMX, a Johnson-Neymann procedure was used. The moderation analysis indicates that there is a positive direct effect between preciseness and LMX (Model 2, Table 3). This relationship is moderated by WUS, as preciseness has a stronger relationship with LMX in small groups (*B* = −0.77^∗∗^, e.t = 0.16 with WUS equal to 2) and as the WUS becomes bigger it smooths and becomes non-significant when the group is bigger than 20 people (Figure 2).

**FIGURE 3:**
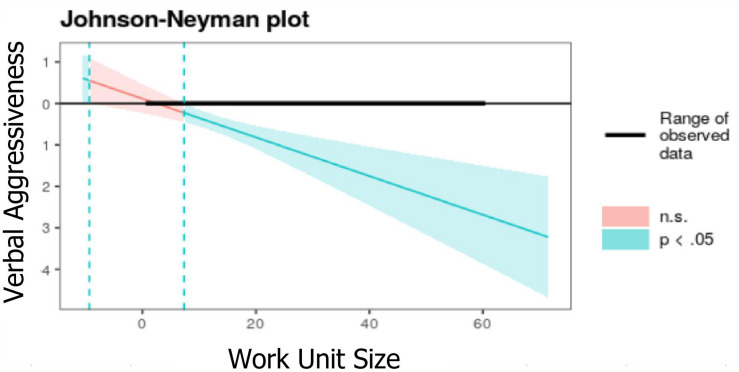
The moderating effect of WUS on the relationship between verbal aggressiveness and LMX.

There is a negative relationship between verbal aggressiveness and LMX (Model 2, Table 3). This relationship is moderated by WUS, when the group size is higher than 13 people (*B* = −0.51^∗∗∗^, e.t = 0.1) and becomes stronger as group size is bigger (e.g., *B* = −1.05^∗∗∗^, e.t = 0.19, with WUS equal to 25 people). Therefore, the WUS enhances the relationship between verbal aggressiveness and LMX (Figure 3).

**FIGURE 4:**
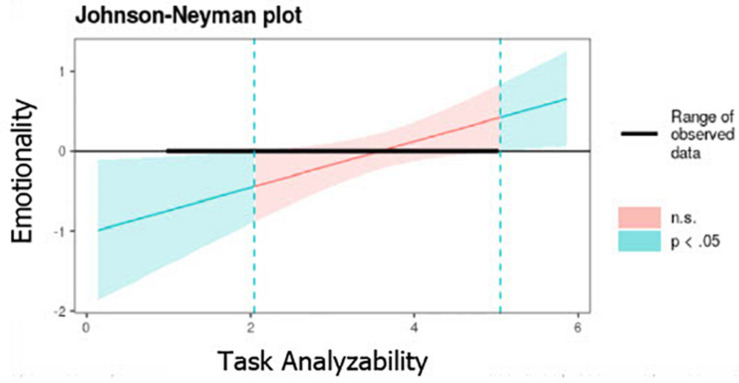
The moderating effect of TA on the relationship between emotionality and LMX.

Even if there is no significant relationship between emotionality and LMX (Model 2, Table 3), there is a negative relationship when TA moderates the relationship between the aforementioned variables. When TA has lower values (values between 1 and 2), the relationship between emotionality and LMX is negative (*B* = −0.75^∗∗^, e.t = 0.34 with TA equal to 1 and smooths as TA increases (*B* = −0.46^∗^, e.t = 0.23 with TA equal to 2 and becomes non-significant when TA is higher than 2. In other words, TA has a damper effect on the relationship between emotionality and LMX (Figure 4).

It is possible that higher levels of TA (values higher than 5) could show a positive relationship between emotionality and LMX but this Hypothesis needs to be tested because in this study TA’s values ranged from 1 to 5.

Model 2 (Table 3) also indicates a significant relation between leader gender and LMX. Men that are leaders have higher levels of LMX (*B* = 0.42, e.t = 0.13) than women that are leaders while controlling for communication styles, age, gender from subordinates.

## Discussion

The results of Model 1 (Table 3), without including the moderating variables, show a significant relationship between four dimensions of a leader’s communication style on the LMX. Expressiveness, preciseness, and questioningness positively affect LMX, while verbal aggressiveness is negatively related. Preciseness shows the most significant impact (β = 0.58, *p* < 0.001). The leader’s ability to construct messages concisely, professionally, and well-structured makes it easier for the subordinate to understand the instructions, expectations, and vision, which strengths LMX. Verbal aggressiveness is the second in impact level (β = −0.35, *p* < 0.05) with a negative impact on the LMX. The communicative behaviors of anger, authoritarianism, derogatoriness, and non-supportiveness weaken the quality of the LMX by causing barriers in the worker and rejection of the leader’s proposal. Questioningness (β = 0.30, *p* < 0.01) and expressiveness (β = 0.24, *p* < 0.01) strengthen the quality of the LMX by showing the leader’s proclivity to stimulate discussion about new ideas, intellectual challenge through conversations transcendent and promote participation and the exchange of opinions by an open, frequent, informal and in a good mood communication.

Emotionality and impression manipulativeness are not significantly related to LMX. In high power distance societies, leader-subordinate relationships tend to be polarized and highly emotional (Hofstede and Hofstede, 2001), which could explain that the emotionality dimension (communicative behaviors that show concern, anxiety, and stress about daily routine issues) is not a relevant factor associated with leadership since it is taken for granted. Along the same lines, the impression manipulativeness in vertical societies can be accepted and deemed necessary to uphold the system’s privileges, status symbols, and prevalence of those “at the top.”

Two sets of hypotheses were proposed to explore the moderating effect of the contextual variables work unit size (WUS) and task analyzability (TA) on the relationship between leader’s communication style and LMX (Model 2, Table 3).

WUS shows a direct negative relationship on LMX (β = −0.01, *p* < 0.05) when controlling all the other variables, which implies that the leader’s ability to maintain high-quality LMX relationships decreases as the WUS grows. When exploring the moderating effect of WUS on the relationship between the leader’s communication style and LMX, two dimensions show significant effect: preciseness (β = −0.03, *p* < 0.05) and verbal aggressiveness (β = −0.05, *p* < 0.001). The other dimensions of the leader’s communication style are not sensitive to the moderating effect of the growth of the WUS. Preciseness and verbal aggressiveness show negative betas. In preciseness, whose effect is naturally positive, it decreases as the work unit grows. Regarding verbal aggressiveness, whose natural effect is negative, as the work unit grows, the effect becomes more negative, damaging the LMX with greater intensity.

The Johnson-Neyman procedure used to measure the moderation analysis indicates that preciseness has a stronger relationship with LMX in small groups (*B* = −0.77^∗∗^, e.t = 0.16) with WUS equal to two, but as the WUS grows, the relationship smooths and becomes non-significant when the group is more extensive than 20 people. Regarding the moderating effect of WUS on the negative relationship between verbal aggressiveness and LMX, it appears when the group is greater than 13 people (*B* = −0.51^∗∗∗^, e.t = 0.1) and becomes more intense as the group grows. In other words, the negative effect of the leader’s verbal aggressiveness on the relationship with subordinates will be most substantial as the group becomes large.

Task analyzability (TA) shows a direct positive relationship on LMX (β = 0.19, *p* < 0.01) when controlling all the other variables, which implies that structured tasks contribute to the quality of the LMX. In other words, when performing structured (low complexity) tasks, the worker maintains good LMX quality with his supervisor.

Although emotionality did not show a significant relationship with LMX (Model 2, Table 3), when the moderating effect of TA is incorporated, it is observed that emotionality is the only dimension that shows a significant positive relationship with LMX (β = 0.29, *p* < 0.05). This means that leader’s emotionality contributes positively to the LMX when TA is in the equation. The Johnson-Neymann procedure shows that TA negatively moderates the relationship (*B* = −0.75^∗∗^, e.t = 0.34) between these variables for values lower than two on a scale of 1 to 5 (low structured tasks and therefore more complex and difficult to perform). In other words, TA has a damper effect on the relation between emotionality and LMX when tasks are low structured (Figure 4). If the worker performs unstructured and complex tasks, the leader’s emotionality (communicative behaviors that show concern, anxiety, and stress about daily routine issues) are less favorable to the LMX.

### Theoretical Implications

Leader’s communication has deserved an extensive attention in the literature from various ontological, epistemological and methodological perspectives (Fairhurst and Connaughton, 2014). The literature widely recognizes the impact of the leader’s communication on organizational results (Phillips et al., 2004). This research uses the construct “leader’s communication style” and a multidimensional six-dimensional model (de Vries et al., 2009, 2011) to fill the existing gap in the literature about how the leader should adapt his communication style to the context to achieve high-quality LMX. An essential contribution of this approach is the empirical evidence that allows us to identify which dimensions of the leader’s communication style contribute to strengthening or weakening the quality of the LMX in the different contexts or situations that leaders face. The theoretical contribution of this study aims to open a research line on leader’s communication from a contingent approach.

High-quality LMX relationships are seen more often when leaders display communication behaviors of openness and support, provide information to reduce ambiguity and make sure their exchanges are timely and high-quality (Campbell et al., 2003; Michael et al., 2005). Additionally, frequent, empathetic, trust-inspiring, kind communication, which shows a willingness to listen, contributes toward a strengthening of the leader-member bond (Mueller and Lee, 2002). The results obtained without considering the contextual variables validate the results reported in the literature, as relationships between four dimensions of the leader’s communication style and LMX were found. Expressiveness, preciseness, and questioningness have a favorable effect on LMX, while verbal aggressiveness is negatively related.

Emotionality and impression manipulativeness are not significant in the context of this study. Peru is a high-power distance and collectivist society (Hofstede, 2016). From his theory of cultural dimensions, Hofstede (Minkov and Hofstede, 2011a, b) propose that in high power distance societies decision structures are centralized, with a high concentration of power and information at the top of the organizational structure. The leader’s behaviors are formal and autocratic. The leader exercises strict supervision, which is satisfactorily perceived by subordinates and contributes to performance and productivity. Superior-subordinate relationships are polarized and often emotional, explaining why emotionality dimension (sentimentality, worrisomeness, tension and defensiveness) is not a trait required for high-quality LMX as it is taken for granted. In the same line, communicative behaviors related to impression manipulativeness (ingratiation, charm, inscrutableness, and concealingness) are accepted as usual and even necessary to maintain the vertical relationship system in the parameters of control, agreeableness, polite and politically correct way.

The existence of a direct relationship between WUS and LMX is a question that remains open due to the contradictory results reported in the literature. The present results coincide with those found by Green et al. (1996), in the sense that the size of the work unit does negatively affect the LMX quality. From a communication perspective, evidence has been found that the impact of leader communication on LMX is sensitive to WUS through the interaction with preciseness and verbal aggressiveness dimensions.

As shown in the results of the first model without moderators, preciseness plays an important positive role in leaders’ communication style. Leaders must be aware that communicating in a direct, concise, structured way contributes to LMX as it facilitates the understanding of the leader’s messages, vision, needs, and expectations, reducing ambiguity and helping subordinates to clarify how to achieve better performance. Additionally, this feature must be modulated considering the size of the work unit. When the group is small, the impact of preciseness is strong, but the effect is diluted as the group grows. Because of this, the leader must look for ways to communicate to strengthen his/her messages, since preciseness will lose its effect as new members join the work unit. According to the results obtained, the positive effect of preciseness disappears when the group exceeds 20 workers, something that contributes to the field of the relationship between communication and leadership.

Verbal aggressiveness is a trait that also plays an important role in the leader’s communication because of its effect on LMX, as could be seen in the results obtained in Model 1. The negative effect on the quality of interpersonal relationships is widely recognized in the literature. The results obtained from the moderation analysis indicate that this trait is sensitive to the size of the work unit. The *per se* negative effect intensifies as the group grows so that when the group exceeds the number of 13 the effect is even more negative. This evidence is a warning light so that leaders are careful with their communications. In small groups, closeness to the leader, access to information, frequency of contact and higher levels of trust can create tolerance for verbal aggressiveness, as the subordinates know the leader better and are better informed regarding the demands and challenges of management. On the other hand, when a group is large, subordinates, who perceive this behavior more negatively, which could be explained by the greater psychological distance from the leader and less information regarding the demands that the leader faces, less tolerate verbal aggressiveness. It is recommended for leaders to consider this when leading large groups; they should refrain from the exteriorization of verbal aggressiveness and increase the frequency of contact with their subordinates so that their LMX quality is not affected.

Regarding *TA* as a context variable, the moderation analysis has shown that it could condition the relationship between the leader communication style and the LMX quality through the emotionality dimension. Although this dimension was no significant in the model without moderation, it becomes significant when it interacts with TA. This interaction becomes evident when a worker carries out low structured tasks (low TA), that is to say, tasks in which there is not a predetermined sequence and the worker needs to think and decide how to solve the problem. The moderation effect is evident in the lowest level of TA (1 to 2, on a 5-point scale). When tasks are low structured, perceiving in the leader communicative behaviors that express concern, anxiety, tension or stress positively contributes to the quality of the LMX as they may be interpreted by a subordinate as sensitivity and understanding of the complexity and difficulty of the challenge that the worker must face. The results also show that it is possible that, in levels of TA higher than 5, the moderation effect appears. This could be understood under the Job Characteristic theory (Hackman and Oldham, 1976) which explains that when a task is well known, structured and not challenging, the leader can contribute to motivation giving significance through his concern, anxiety or tension. This issue needs to be explored in future studies.

The results obtained in this research contribute to expanding the understanding of the relation between LMX and communication from a contingent perspective. A leader’s communication style is a vital instrument in the construction of the LMX bond and leaders should be aware that the effect of their style could be different according to the context. The size of the work unit and the task analyzability are at least two context variables that they should consider when interacting with their followers.

### Management Implications

These findings have managerial implications as they confirm that leaders possess, in their own communication styles, a tool for improving the quality of their relationships with subordinates and favoring their leadership. The subjects of our study are white-collar professionals, most of whom have completed higher education. Managing personnel of these characteristics is a challenge, so the leader must consider the implications of his/her communication style on LMX and the achievement of results.

Followers appreciate that their leader communicates openly and loquacity, somewhat informally and in a good mood, because these behaviors promote trust and reduce unnecessary barriers. Additionally, they appreciate that their leader communicates in a precise, concrete, direct, structured way without going around the bush or presenting irrelevant information. Due to information and communication technologies, we are currently exposed to an overload of information. We have to pay attention to emails, telephone, meetings, documents. If we consider that the sample subjects are knowledge workers, they are professionals whose production are ideas, solutions, proposals, initiatives that others must value to be implemented. They need their leaders to be good communicators with high levels of expressiveness, precision, and questioningness. Encouraging dialog, exchanging opinions, questioning ideas to find new approaches, and thinking “outside the box” are communicative behaviors that will favor LMX and performance.

Conversely, the verbal aggressiveness, which is observed in communicative behaviors such as the open expression of displeasure or anger about issues or people, irritability, authoritarianism, is rejected and affects creating good LMX relationships. Leaders with highly aggressive verbal behaviors tell people what to do and expect their obedience; when asking for something, the tone of voice is demanding. They manifest little respect for others’ opinions, discourage dialog, humiliate, hurt feelings, and make others look like fools. The subordinates feel that the leader neither gives attention to them nor understands their problems or needs, offers little support, and treats people in a distant and impersonal way. They will be less likely to approach the leader to inquire or report, which will reduce the possibility of high-quality LMX and affect team performance.

As the group grows, the favorable effect of preciseness in creating the LMX fades, possibly because the leader will be less likely to interact one-on-one with each member of the team. That is why, to manage the team and achieve results efficiently, the leader must reinforce precision by using communication techniques such as reinforcement (sending messages through several channels simultaneously). Communicating a message face to face and then sending it by email will be better than sending it to the entire large group by instant messaging. Being visible frequently could let them know the leader’s communication style through virtual meetings, podcasts, videos will also be favorable. Using written channels such as the institutional magazine, the Web, flyers, or other documents could raise the perceived preciseness.

When the leader manages large groups, the negative effect of verbal aggressiveness explained above increases as the group grows and should modulate these communicative behaviors, reducing them to a minimum. When the leader has few collaborators, the continuous daily work creates closeness and mutual knowledge that can help understand and even forgive the aggressive behaviors of the leader. However, when the group is large, the worker’s infrequency with the leader makes his/her aggressive verbal behaviors hit much more intensely, thus deteriorating the LMX and affecting his/her chances of achieving good management results.

Professional or knowledge workers usually perform low analyzability (complex) tasks. In this context, the leader’s communicative behaviors associated with emotionality deteriorate the LMX. Leaders are under pressure for their position’s responsibilities, which can lead them to express concern, anxiety, and stress about daily routine issues. Furthermore, as a mechanism for protecting against dissenting opinions or criticisms, the leader copes poorly with critical remarks. These emotional behaviors will affect the quality of the LMX because they will generate rejection. The knowledge worker must think about how to solve the tasks, which are complex in themselves, and seeing the tense and anxious leader does not help them.

The LMX theory is based on the dynamic co-creation of the leader-follower link, in which both contribute for better or for worse. This is a complex dynamic, in which the context determines the behavior of both (Uhl-Bien, 2021). It is hoped that the findings of this research can be incorporated into management training programs at universities and leadership training institutions.

### Limitations and Future Lines of Research

This study contributes by deepening the understanding of which dimensions of leader communication style should be modified to improve the quality of the LMX relation according to the context, contribution that could be useful in leadership training at business schools.

While our study’s findings have important implications for the theory and practice of leadership, as with all studies it has a number of limitations. First, this contribution should be interpreted taking into consideration that it was obtained through a sample of subjects (subordinates) who all have advanced educational degrees and ample work experience as managers, and therefore, the results may not be generalized. This professional group, known in the literature as “white collar” workers, possess characteristics that limit generalization to the entire working population, and therefore, it is necessary for future studies to replicate the research with a more representative sample. Second, and in line with the first limitation, the sample size is small, and the study is cross-sectional, so we may consider this as a pilot study. Third, our data represent a single country, which prevents the results to be generalized worldwide.

These limitations open up avenues for future research. Further research may identify other applications by studying samples of other strata in the working population. From a contingency perspective, the line of research on leader communication and its relationship with LMX offers multiple paths. There are many contextual characteristics and elements that the literature shows have an impact on workgroups, and these could be considered for future research to contribute to management practices. Future lines of research should include not only the impact of virtuality, digitization, artificial intelligence, but the different ways the job is executed, either physically, remotely or a combination of the two, being the last two accelerated by the forces that have been unleashed by the COVID-19 pandemic. As another future line of research, it could be interesting to see the differences in other countries. The cultural context may influence the moderating effect of the different variables. Additionally, the fact that the leader and subordinate belong to different cultures or ethnicity may have an influence on their communication style and LMX.

### Conclusion

There is not much research on how context variables affect communication behaviors in the business environment. The results obtained in this research add value from two perspectives. It’s important to know what traits of a leader’s communication must be modulated according to WUS and TA., but it is also good to know which ones do not need so much attention. This may help leaders to be more aware of their communicative behaviors in order to focus on those that could help or harm the results of leadership on a day-to-day basis.

From an academic perspective, this paper contributes to the field of organizational behavior, having presented a study on communication from a contingency approach. Six dimensions of a leader’s communication style have been explored because they influence interpersonal relationships with subordinates; they are ever-present as they are the constitutive elements of the communication style itself (de Vries et al., 2011; Bakker-Pieper and de Vries, 2013). Leaders must be aware of the impact that their communication styles have on their effectiveness to build high-quality leader-member relationships. Leaders should be sensitive to context factors as WUS and TA and modulate the way they communicate with subordinates to enhance LMX. For large groups, the leader must be aware of his/her preciseness and verbally aggressiveness, when communicating. The positive effect of preciseness perceived by subordinates on a leader’s messages will decrease as the group grows. Additionally, there must be a reduction of verbal aggressive behaviors because their negative effect is more harmful as a group grows. It is important also to consider the degree of TA to be carried out by the workers to interact sufficiently with each one. When the task to be carried out is low structured (low analyzability), an increase of emotionality may contribute to building high-quality LMX relationships. Perceiving the leader’s tension, anxiety, worrisomeness, and defensiveness could enhance LMX as the subordinate understands that his/her leader understand the complexity and challenge of the task he/she must face.

## Data Availability Statement

The raw data supporting the conclusions of this article will be made available by the authors, on request, without undue reservation.

## Author Contributions

Both authors listed have made substantial, direct and intellectual contributions to the work, and approved it for publication.

## Conflict of Interest

The authors declare that the research was conducted in the absence of any commercial or financial relationships that could be construed as a potential conflict of interest.
